# Base Rates of Depressive Symptoms in Patients with Coronary Heart Disease: An Individual Symptom Analysis

**DOI:** 10.1371/journal.pone.0156167

**Published:** 2016-05-26

**Authors:** Sebastian Kohlmann, Benjamin Gierk, Alexandra M. Murray, Arne Scholl, Marco Lehmann, Bernd Löwe

**Affiliations:** 1 Department of Psychosomatic Medicine and Psychotherapy, University Medical Center, and Schön Clinic Hamburg-Eilbek, Hamburg, Germany; 2 Department of Psychiatry, Asklepios Clinic North Ochsenzoll, Hamburg, Germany; Istituto Superiore di Sanità, ITALY

## Abstract

**Background:**

Major depression is common in coronary heart disease (CHD) but challenging to diagnose. Instead of focusing on the overall diagnosis of depression, base rates of depressive symptoms could facilitate screening and management of psychopathology in CHD. The present study investigates the frequency of individual depressive symptoms in CHD and their impact on cardiac and subjective health.

**Methods:**

In total, 1337 in- and outpatients with CHD were screened for depressive symptoms with the Patient Health Questionnaire-9 (PHQ-9) at three different cardiac treatment sites. Tables stratified by age and gender were designed to illustrate base rates of depressive symptoms. Multiple regression analyses adjusted for sociodemographic and clinical data were conducted to test associations between individual depressive symptoms and quality of life as well impairment caused angina pectoris and dyspnea.

**Results:**

During the last 14 days, more than half of patients reported a loss of energy (74.9%, 95% Confidence Interval (CI): 70.6–79.2), sleeping problems (69.4%, 95% CI: 64.9–74.0), loss of interest (55.7%, 95% CI: 50.8–60.7). In contrast, psychomotor change (25.6%, 95%CI: 21.3–30.0), feelings of failure (21.9%, 95%CI: 17.7–26.0), suicidal ideations (14.1%, 95%CI: 10.7–17.6) were less frequently reported. Depending on the outcome, only particular depressive symptoms were highly associated with low quality of life and impairment caused by angina pectoris and dyspnea. Loss of energy was the only depressive symptom that reliably predicted all three outcomes.

**Conclusions:**

Depressive symptoms in CHD are frequent but vary widely in terms of frequency. Findings underline the differential effects of individual depressive symptoms on cardiac health. Presented base rates of depressive symptoms offer clinicians a new way to judge the severity of individual depressive symptoms and to communicate individual PHQ-9 profiles with patients with respect to gender, age, cardiac symptoms and quality of life.

## Introduction

Depression in patients with coronary heart disease (CHD) is a complex condition with a poorly understood etiology [1, 2]. Thus, diagnosing major depression within this patient population is clinically challenging and available depression treatments show only modest effects in patients with CHD [3–5].Therefore, new approaches to diagnose and treat, but also to conceptualize depression in CHD are needed [6]. A symptomatic approach to depression that investigates individual depressive symptoms has recently gained more awareness and could overcome the challenge to understanding psychopathology in cardiac patients [6].

Indeed, a closer look at several studies reveals that rather than the whole construct of depression, only individual symptoms of depression are stable predictors of worse cardiac outcome in the long term [7–9]. Hoen et. al. (2010) found appetite, fatigue, sleeping problems and depressed mood were able to predict cardiac events [9]. Denollett et al. (2013) reported that somatic symptoms of depression (i.e. fatigue) and hopelessness predict mortality in patients with CHD [8]. Warrings et al. (2012) showed that changes in appetite predict mortality in patients with chronic heart failure [10]. Instead of focusing on depression as a whole entity, research that investigates the prognostic value of individual depressive symptoms themselves might be a more appropriate approach to understand why psychological distress leads to increased risk of cardiac morbidity and mortality.

A symptomatic approach to depressive psychopathology appears to be useful from not only a scientific perspective but also a clinical one: In fact, randomized-controlled trials in patients with CHD that targeted major depression with state-of-the-art depression treatment (i.e., antidepressants or cognitive behavioral therapy) showed only small effects on depression [11–13]. These disappointing findings of the first large randomized controlled trials might be due to treatment-related difficulties that are specific for studies that focused on depression as a homogenous condition. By definition, however, the diagnosis of major depression is based on a heterogeneous subset of symptoms that can vary between individuals (e.g., appetite loss versus increased appetite; insomnia versus hypersomnia) and within individuals (e.g., psychomotor agitation on most days and great loss energy on other days) [14]. In patients with CHD, the diagnosis of major depression is especially difficult as somatic symptoms of depression overlap with cardiac symptoms: For example, fatigue and sleeping problems have been found to be more prevalent than angina pectoris in outpatients with CHD [15].

In addition to the challenge of diagnosing depression in cardiac patients, a recent qualitative study indicates that health care providers often perceive depression as a diagnosis without a clear management strategy (in contrast to standardized cardiac procedures) [16]. The label of depression is also associated with perceived stigma which might be a barrier for nurses and cardiologists to communicate the diagnosis. Moreover, mental health care is a rare resource in most cardiac settings and patients are rarely referred to mental health specialists [17, 18].

Taken together, these findings could be an explanation why depression screening in patients with CHD has not yet been found to be beneficial in terms of detecting rates or depression management [18, 19]. The potential therapeutic value of depression screening is based on the clinician’s willingness to openly discuss the screening results and then, how the patient copes with the psychiatric diagnosis [20]. Thus, there is a need to establish easy-to-access and time-effective tools for clinicians in busy cardiac settings to increase awareness of the risk factor depression and to facilitate subsequent patient-oriented communication [21, 22]. Norm-tables that illustrate the natural occurrence of individual depressive symptoms could prompt clinicians to openly discuss profiles of burdensome depressive symptoms with cardiac patients.

From a research perspective, a symptomatic approach is promising to understand the psychopathology of depression in CHD. On the other hand, from a clinical perspective, simple base rates of depressive symptoms form the basis of an easy to use patient-oriented tool that helps clinicians in busy cardiac settings discuss emotional distress with patients. Whereas the prevalence of the construct depression is well investigated, data on the frequency and range of individual depressive symptoms has yet not been studied. Consequently, it is difficult to state how common individual depressive symptoms in patients with CHD are, what depressive symptom burden is in normal range and whether this is dependent on socio-demographic or clinical characteristics. Therefore, the aim of the present study is, first, to estimate the range and frequency of individual depressive symptoms and their relationship with age and gender and, second, to analyze their unique associations with quality of life and core cardiac symptoms (i.e. dyspnea and angina pectoris).

## Methods

### Study design, patients and procedure

The present study used cross-sectional data from the baseline assessment of the DEPSCREEN-INFO trial (ClinicalTrials.gov, Identifier: NCT01879111). DEPSCREEN-INFO is a randomized controlled trial which examines two different depression screening strategies in patients with CHD or hypertension. The present study focusses on patients with CHD. Thus, patients were included if they were diagnosed as having a CHD by a cardiologist, if patients were aged above 18 years and had sufficient language skills (German). Patients were excluded if they met one of the following exclusion criteria: life threatening health status, severe somatic or psychiatric disorder that required urgent treatment, severe cognitive, motor or visual difficulties, or no provision of written informed consent. Ethics were approved by the Medical Association, Hamburg, Germany (No. PV3845/ Ethics’ approval date: September 1st 2011) and written informed consent was obtained from all patients. Between October 2011 and October 2013, 2807 patients with known CHD were approached consecutively from three study sites in Hamburg, Germany; i.e., a large cardiology outpatient center, the cardiology outpatient clinic and an inpatient ward of the University Heart Centre. Out of 2807 patients screened for eligibility, 1337 patients with clinically confirmed CHD were eligible to participate. Reasons for exclusion were: not providing informed consent (n = 1019), early discharge (n = 205), severe somatic disorder that needed urgent treatment (n = 93), language difficulties (n = 87) and severe cognitive, motor or visual difficulties (n = 66). Eligible patients were invited to complete a set of questionnaires while waiting for their consultation.

### Measures

Depressive symptoms were assessed with the Patient Health Questionnaire-9 (PHQ-9) [23]. The PHQ-9 consists of nine items reflecting the DSM-IV criteria for major depression (i.e. loss of interest, feeling down, sleeping problems, loss of energy, appetite change, feelings of failure, trouble concentrating, psychomotor change, suicidal ideations). The questionnaire assesses the frequency (‘not at all’; ‘several days’; ‘more than half the days’; ‘nearly every day’) of the symptoms over the past two weeks. The PHQ-9’s psychometric properties have been shown to be good in several validation studies [24–26]. The American Heart Association Science Advisory recommended the PHQ-9 for depression screening in patients with CHD [27].

Health-related quality of life was measured with the EuroQol-5D (EQ-5D) which is a simple generic measure that summarizes health-related quality of life in a single index [28]. Current health state in 5 dimensions (mobility, self-care, usual activities, pain/discomfort, and anxiety/depression) is assessed on a 3-point scale (“no problems”, “moderate problems”, “extreme problems”). A population based index (EQ-5D-index) is calculated to reflect the current health status Score ranges from -0.205 to 0.999; lower scores represent a worse quality of life. In patients with CHD an average score range between 0.76 and 0.82 has been reported [29, 30].

The core cardiac symptom of CHD is angina pectoris. With increasing cardiac disease severity (such as the development of chronic heart failure) dyspnea is frequently observed. The impairment caused by dyspnea and angina pectoris was rated according to the New York Heart Association (NYHA) class and Canadian Cardiology Society (CCS) class, respectively. Both classification systems are established markers of the functional severity of heart diseases [31, 32]. According to the impairment level, four classes are determined ranging from ‘no impairment at all’ to ‘impairment even at resting’. With increasing symptom impairment the classes are rated higher. As clinical characteristics, treatment setting, history of myocardial infarct and bypass surgery were assessed. Cardiac risk factors were assessed including hypertension, diabetes and dyslipidemia. In addition, information based on self-report regarding current smoking behavior, family history, and obesity were collected.

### Statistical analyses

First, base rates to estimate the range and frequency of depressive symptoms for the whole sample were analyzed. The sample was then stratified by age and gender as increased rates of depression have been reported for younger and female individuals [33, 34]. For clinical purposes and in line with epidemiological studies on depression and cardiac outcomes, the age cut-off was set to 65 years [35]. Cohen’s d effect sizes were calculated to test differences in individual depressive symptoms between genders.

Second, three multiple hierarchical regression analyses were performed to test the predictive value of each depressive symptom on dyspnea (NYHA class), angina pectoris (CCS class) and health related quality of life (EQ-5D). To adjust for confounding variables, predictors were entered block wise. First, socio-demographic, second clinical characteristics, third cardiac risk factors and, then nine individual depressive symptoms were entered in the final step to test the contribution of each depressive symptom on NYHA class, CCS class and quality of life. Variance inflation factors were calculated for each model to test for multicollinearity among predictors. To check whether depressive symptoms were robust predictors, all models were cross-validated using random split-half samples. Given that multiple tests were performed, a false discovery rate approach (Benjamini-Hochberg procedure) was applied when judging the significance of each predictor to reduce the risk of alpha inflation [36].

Given that our sample corresponds to the baseline sample of patients with CHD from the DEPSCREEN-INFO trial, sample size was fixed for the analyses of this study. Therefore, we performed a post-hoc power-analysis to determine the power of the multiple regression analyses in this study. Given a sample size of n = 1337 patients, our analyses were sufficiently powered (1-*β* = 0.89) to detect even small effects on cardiac symptom burden and quality of life when testing 22 predictors (4 socio-demographic factors, 3 clinical characteristics, 6 cardiac risk factors, 9 depressive symptoms) in linear regression models [37].

Missing data was less than 2% on every PHQ-9 item. Thus, missing data were not imputed and all available information was used (pairwise deletion). Analyses were performed using SPSS Version 22.0 (Chicago Inc).

## Results

### Sample

Information regarding the patient characteristics of the whole patient sample as well as age and gender stratified subsamples are provided in Table 1.

**Table 1:** Characteristics of 1337 patients with CHD.

	All patients	Aged 65 years or under		Aged older 65 years		Differences
Group		A	B	C	D	
		Male	Female	Male	Female	
	N = 1337	n = 390	n = 127	n = 564	n = 256	
**Sociodemographics**						
Age, mean (SD), years	67.5 (10.4)	56.9 (6.8)	56.4 (7.3)	73.9 (5.1)	74.9 (5.7)	None
Female, no (%)	383 (28.6)	0 (0.0)	127 (100)	0 (0.0)	256 (100)	None
≥ 10 years of formal education, no (%)^a^	626 (48.3)	211 (54.5)	65 (53.3)	265 (48.7)	85 (35.0)	A,B,C *>* D *
Employed, no (%)^b^	264 (20.2)	198 (50.8)	50 (40)	15 (2.7)	1 (0.4)	A,B *>* C,D *
**Clinical characteristics, no (%)**						
Myocardial infarct^c^	640 (48.7)	219 (56.7)	53 (42.4)	266 (48.1)	102 (40.6)	A > B,C,D *
Bypass surgery^d^	369 (28.2)	85 (22.3)	21 (16.9)	209 (37.6)	54 (21.8)	A,C > B,D *
In-patient treatment	370 (27.7)	94 (24.1)	29 (22.8)	169 (30.0)	78 (30.5)	None
**Cardiac risk factors no (%)**						
Hypertension^e^	954 (72.4)	280 (72.2)	94 (75.2)	394 (70.6)	186 (75.3)	None
Diabetes^f^	395 (30.0)	113 (29.1)	42 (33.6)	170 (30.5)	70 (28.3)	None
Dyslipidemia^g^	901 (68.3)	276 (71.1)	90 (72.0)	351 (62.9)	184 (73.9)	A,B,D > C *
Smoking^h^	245 (18.5)	114 (29.5)	34 (27.2)	61 (10.9)	36 (14.4)	A,B > C,D *
Obesity^i^	419 (32.1)	147 (38.5)	56 (45.9)	150 (27.0)	66 (26.8)	A,B > C,D *
Family history^j^	680 (51.4)	212 (54.5)	82 (65.6)	241 (43.0)	145 (58.0)	A,B,D > C *
**Dyspnea, no (%)**^k^						None
NYHA Class I	538 (41.2)	188 (48.3)	43 (35.0)	228 (41.9)	79 (31.6)	
NYHA Class II	413 (31.6)	110 (28.3)	45 (36.6)	165 (30.3)	93 (37.2)	
NYHA Class III	266 (20.4)	72 (18.5)	27 (22.0)	111 (20.4)	56 (22.4)	
NYHA Class IV	89 (6.8)	19 (4.9)	8 (6.5)	40 (7.4)	22 (8.8)	
**Angina pectoris, no (%)**^l^						None
CCS Class I	742 (57.1)	225 (58.3)	58 (47.5)	338 (62.0)	121 (49.0)	
CCS Class II	243 (18.7)	70 (18.1)	27 (22.1)	96 (17.6)	50 (20.2)	
CCS Class III	159 (12.1)	47 (12.2)	14 (11.5)	66 (12.1)	32 (13.0)	
CCS Class IV	156 (12.0)	44 (11.4)	23 (18.9)	45 (8.3)	44 (17.8)	
**Quality of life, mean (SD)**^m^	0.79 (0.2)	0.82 (0.2)	0.74 (0.2)	0.80 (0.2)	0.75 (0.2)	None
**Depression, mean (SD)**	5.5 (4.6)	5.9 (5.0)	6.8 (4.7)	4.8 (4.3)	5.9 (4.4)	None

Note

* Differences tested with ANOVA using Bonferroni-adjustment, significant if *p <* .008.

^a^ missing data n = 41

^b^ missing data n = 32

^c^ missing data n = 22

^d^ missing data n = 28

^e^ missing data n = 19

^f^ missing data n = 19

^g^ missing data n = 17

^h^ missing data n = 16

^i^ missing data n = 16

^j^ missing data n = 13

^k^ missing data n = 31

^l^ missing data n = 37

^m^ missing data n = 51

### Frequency and range of depressive symptoms and their associations with gender and age

The frequency of depressive symptoms varied widely between symptoms. Symptoms that patients reported as being present on at least several of the last 14 days were prevalent in the following descending order: loss of energy 74.9% (95% Confidence Interval (CI): 70.6–79.2), sleeping problems 69.4%, (95% CI: 64.9–74.0), loss of interest 55.7% (95% CI: 50.8–60.7), trouble concentrating 39.3% (95%CI: 34.4–44.1), feeling down 38.0% (95%CI: 33.2–42.8), appetite change 37.7% (95%CI: 32.8–42.5), psychomotor change 25.6% (95%CI: 21.3–30.0), feelings of failure 21.9% (95%CI: 17.7–26.0), suicidal ideations 14.1% (95%CI: 10.7–17.6).

**Fig 1:**
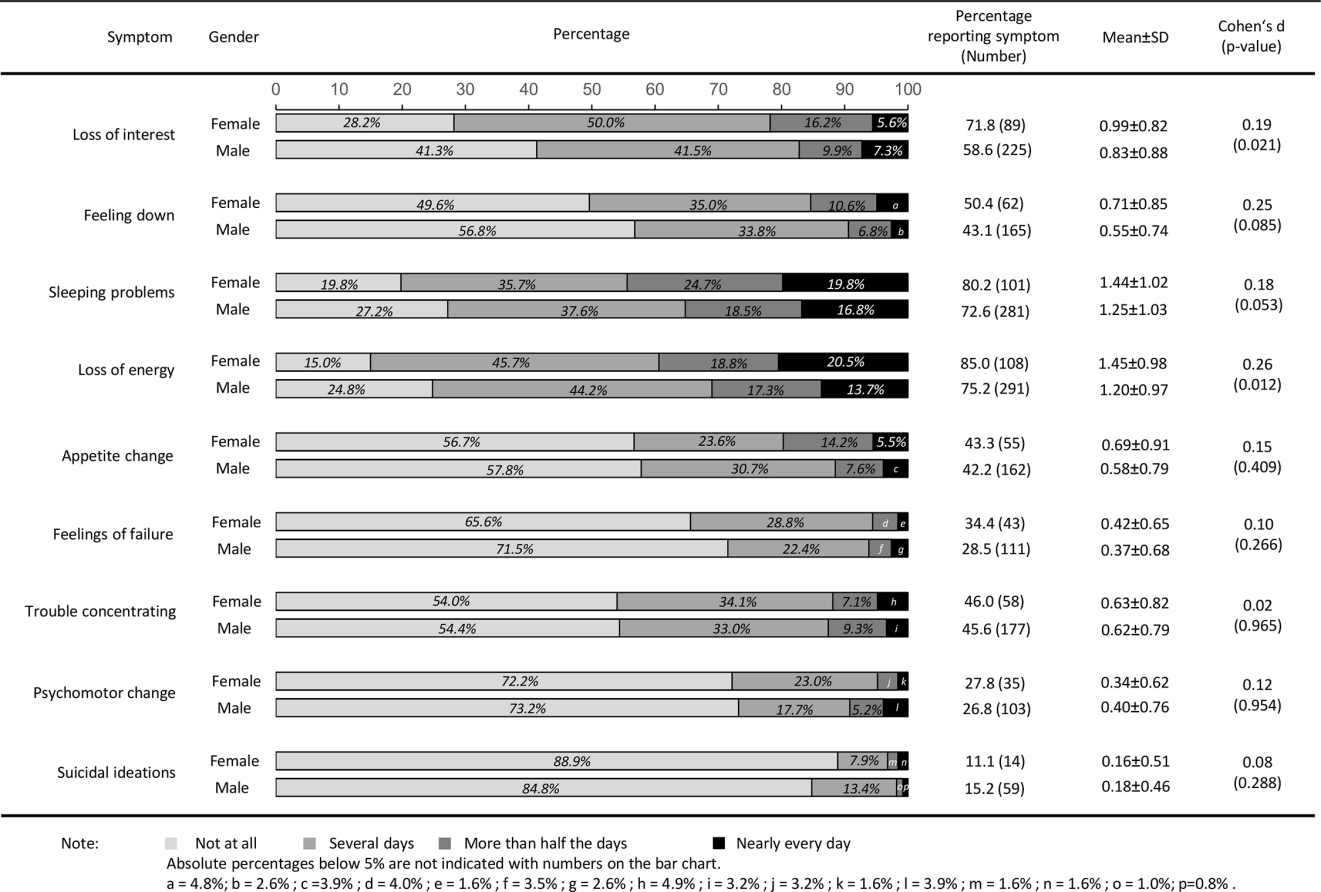
Depressive symptoms of 127 female and 390 male patients with coronary heart disease aged 65 years or under.

**Fig 2:**
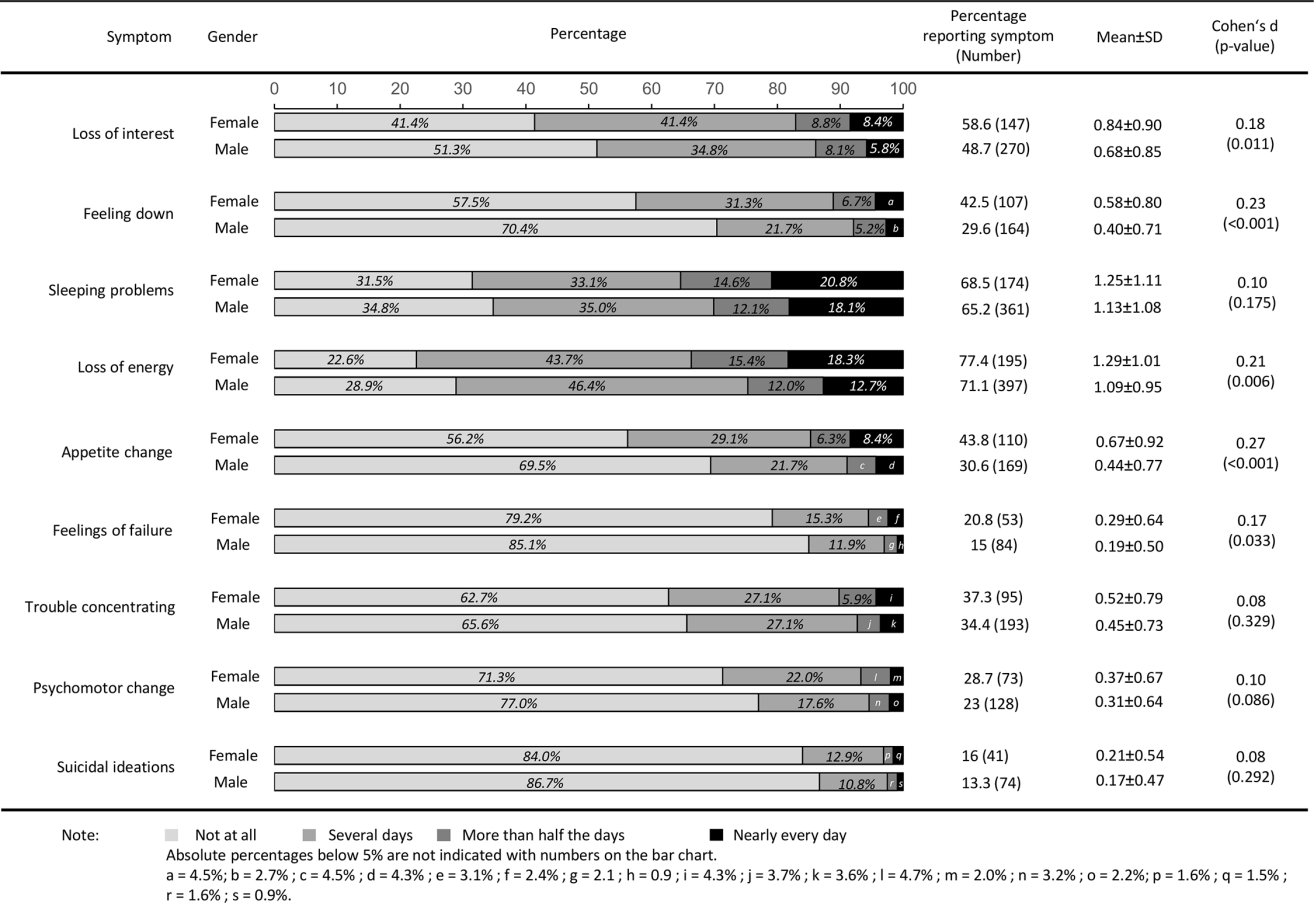
Depressive symptoms of 256 female and 564 male patients with coronary heart disease aged over 65 years.

The frequency of depressive symptoms stratified by age and gender are shown in Fig 1 and Fig 2. Within these subsamples, depressive symptoms also varied widely in terms of their frequencies. Differences on depressive symptoms between genders were small as indicated by Cohen’s d effect sizes. Compared to male patients, female patients aged 65 years or under reported higher symptom burden in terms of loss of interest and loss of energy (*p* = 0.021 and *p* = 0.012, respectively). In patients aged over 65, more differences between genders were indicated: Female patients aged over 65 reported loss of interest (*p* = 0.011), feeling down (*p*<0.001), loss of energy (*p* = 0.006), appetite change (*p*<0.001) and feelings of failure (*p* = 0.033) more frequently than male patients aged over 65.

### Associations between depressive symptoms with dyspnea, angina pectoris and quality of life

**Fig 3:**
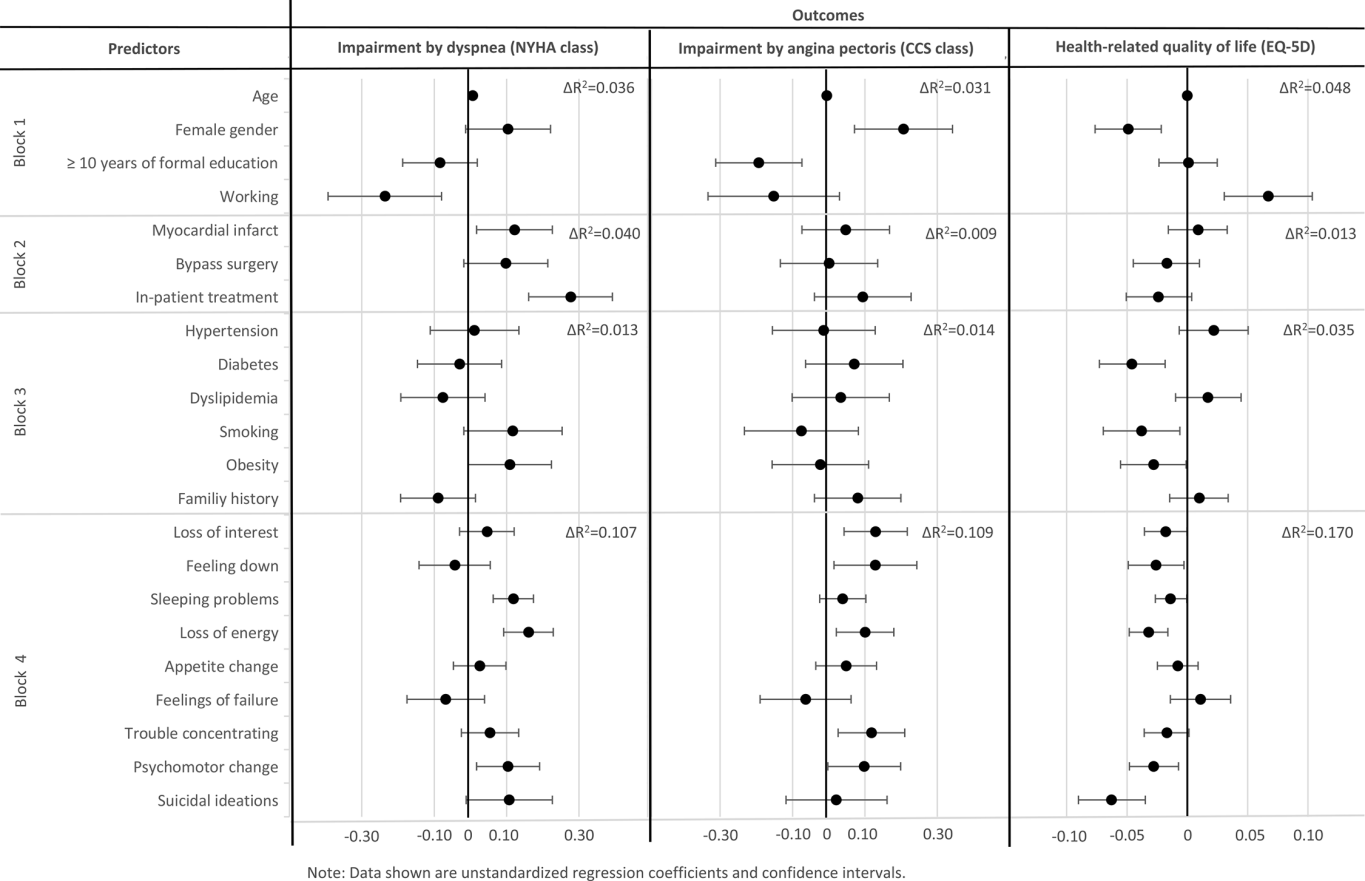
Multiple regression models predicting dyspnea burden(NYHA) class, angina pectoris burden (CCS) class and quality of life (EQ-5D).

To visualize differential effects of individual depressive symptoms on NYHA class, CCS class and EQ-5D, the unstandardized regression coefficients and confidence intervals are displayed in Fig 3. Predictors were entered block-wise: First, demographics, second, clinical characteristics, third, cardiac risk factors. After adjusting for these covariates, depressive symptoms were entered in the final step to determine their unique contribution to health-related quality of life (EQ-5D), and impairment caused by angina pectoris (CCS class) and dyspnea (NYHA class). The average variance inflation factor was 1.34 indicating no multicollinearity among predictors.

The overall regression model predicting NYHA class accounted for 19.7% of the total variance (F = 11.99, *p*<0.001). After adjusting for covariates, depressive symptoms accounted for 10.7% variance in NYHA class (*F* = 16.06 *p*<0.001). The standardized beta coefficients indicated that in addition to not being currently employed (*β* = -0.10, *t* = -2.96, *p* = 0.003), having had a myocardial infarction (*β* = 0.07, *t* = 3.30, *p* = 0.021), and being in an in-patient setting (*β* = 0.13, *t* = 4.71, *p*<0.001), higher impairment due to dyspnea was independently predicted by the following depressive symptoms: sleeping problems, (*β* = 0.14, *t* = 4.23 *p*<0.001), loss of energy (*β* = 0.17, *t* = 4.63, *p*<0.001), and psychomotor change (*β* = 0.07, *t* = 2.35, *p* = 0.019). Sleeping problems and loss of energy remained stable significant predictors of NYHA class when a sensitivity analysis using a random split-half sample was performed. These depressive symptoms were also significant when a false discovery rate approach was applied.

The overall regression model predicting CCS class accounted for 16.2% of the total variance (*F* = 9.50, *p*<0.001) of which depressive symptoms accounted for 10.9% (*F* = 15.58, *p*<0.001) after adjusting for covariates. Beta coefficients indicated that in addition to next to female gender (*β* = 0.09, *t* = -2.99, *p* = 0.003) and having a lower level of formal school education (*β* = -0.09, *t* = -3.17, *p* = 0.002), higher impairment caused by angina pectoris was independently predicted by the following depressive symptoms: loss of interest, (*β* = 0.11, *t* = 2.92 *p* = 0.004), feeling down (*β* = 0.09, *t* = 2.21, *p* = 0.028), loss of energy (*β* = 0.09, *t* = 2.49, *p* = 0.013) and trouble concentrating (*β* = 0.08, *t* = 2.51, *p* = 0.012). Loss of interest, feeling down and trouble concentrating remained stable significant predictors of CCS class when a sensitivity analysis using a random split-half sample was conducted. When the false-positive rate approach was applied, however, only loss of interest remained a significant predictor.

The overall regression model accounted for 26.7% of the total variance in EQ-5D (*F* = 17.53, *p*<0.001). After adjusting for covariates, depressive symptoms accounted for 17% (*F* = 27.38, *p*<0.001). Beta coefficients indicated that in addition to female gender (*β* = 0.10, *t* = -3.50, *p*<0.001), and not being currently employed (*β* = 0.12, *t* = 3.60, *p*<0.001), having diabetes (*β* = -0.09, *t* = -3.33, *p* = 0.001), being a smoker (*β* = -0.07, *t* = -2.35, *p* = 0.019) and being obese (*β* = -0.06, *t* = -2.01, *p* = 0.045), lower quality of life was independently predicted by the following depressive symptoms: loss of interest (*β* = -0.09, *t* = -2.21, *p* = 0.027), feeling down (*β* = -0.07, *t* = -2.00, *p* = 0.046), sleeping problems (*β* = -0.07, *t* = -2.19, *p* = 0.029), loss of energy (*β* = -0.14, *t* = -3.89, *p*<0.001), psychomotor change (*β* = -0.08, *t* = 2.73, *p* = 0.006) and suicidal ideations (*β* = -0.14, *t* = -4.50, *p*<0.001). Loss of interest, sleeping problems, loss of energy, psychomotor change and suicidal ideations remained stable significant predictors of low quality of life when a sensitivity analysis using a random split-half sample was calculated. When an approach to reduce the false-positive rate was taken, only loss of energy, psychomotor change and suicidal ideations were significant predictors.

## Discussion

The present study uses a symptomatic approach to examine depression in heart disease and, therefore, investigated individual depressive symptoms in patients with CHD. Results demonstrate that depressive symptoms are common but the frequency of each symptom varies widely. Presented base rates of depressive symptoms will allow clinicians to judge the severity of individual depressive symptoms and interpret a patient’s PHQ-9 profile with respect to gender, age, cardiac symptoms and quality of life. Depression explained substantial amount of variance in multivariate models predicting quality of life and impairment caused by dyspnea and angina pectoris. However, only certain depressive symptoms are stable predictors. Taken together, these findings underline the differential effects of depressive symptoms on health burden and highlight the importance of focusing on individual depressive symptoms in patients with CHD.

The average depression score in our sample was are comparable with other studies investigating depression in patients with CHD [38]. Results of the present study, however, clearly show that the prevalence of individual depressive symptoms varies widely: More than two-thirds of patients reported a loss of energy and sleeping problems during the last two weeks, whereas feelings of failure and suicidal ideations were reported by every fifth and seventh patient, respectively. As the frequency of depressive symptoms varies widely, results support the assumption that depression in patients with CHD is defined as a heterogeneous subset of symptoms rather than a homogeneous condition. The assumption that major depression is clinically not perceived as a condition with specific symptoms is also reflected by studies showing that the diagnosis of major depression in primary care rather depends on the general practitioners subjective concept of depression than on the DSM-defined symptoms of depression [39, 40]. So far, no study has investigated how major depression is perceived or diagnosed in routine cardiac care.

Interestingly, the ranking of the frequency of symptoms in the current study is comparable to the general population but symptom frequency is different [41]. While certain symptoms (such as feelings of failure or suicidal ideations) are almost equally prevalent as in the normal population, symptoms like loss of energy or sleeping problems appear to be more frequent: Indeed, results show that the latter two depressive symptoms are more likely to be present than absent in patients with CHD. A comparative analysis between patients with CHD, patients with unipolar major depression, and individuals from a norm population would be a vital approach to judge whether certain depressive symptoms are more specific to CHD.

Regarding the heated debate on depression screening in CHD [19], the present findings highlight how frequently depressive symptoms burden CHD patients and support a patient-centered screening strategy that focusses on individual PHQ-9 profiles rather than an overall sum score [6]. The presented norm tables of depressive symptoms could be used to compare individual PHQ-9 profiles and, either, to normalize patient’s worries about certain symptoms (i.e. sleeping problems) or to discuss stigmatized symptoms (i.e. suicidal ideations). This way patients with CHD might better understand their symptoms and be more motivated to follow a referral to a mental health specialist [42].

The presented base rates of depressive symptoms also highlight that symptoms like sleeping problems, loss of energy and interest are very frequent in patients with CHD. These symptoms in particular have repeatedly been found to reduce the efficacy of depression treatment and are predictors of chronic major depression [43–45]. This finding might explain the modest treatment effects on depression in this cardiac patient population. A re-analysis of well-established treatment trials would be a promising investigation to test whether specific treatment approaches had effects on individual burdensome depressive symptoms [3, 12, 13, 46]. This way effective treatment strategies could be developed to reduce the frequent health burden of depressive symptoms in patients with CHD.

Psychometrically, depression in cardiac patients appears to have a two-factor-structure that distinguishes a somatic-affective and a cognitive-affective factor [2]. In terms of external validity, the somatic-affective factors predicts worse cardiac outcomes and ‘somatic depression’ has already been suggested to be a cardio-toxic disorder [47–49]. However, the cognitive-affective factor has also been shown to predict cardiac cardiovascular mortality and recurrent cardiovascular events [8, 50]. In brief, differentiating between somatic and affective factors might be useful when conceptualizing depression psychometrically. The prognostic value of these two factors, however, is mixed in the literature [51–53]. Our results support these findings: both somatic and affective symptoms of depression were associated with impairment caused by dyspnea and angina pectoris. Thus, the diagnostic distinction between somatic versus affective depression provides little clinical insights how to tackle the challenge of diagnosing and treating psychological distress in patients with CHD.

Promising RCTs that followed a patient-centered approach and focused on stress-management and patient preferences were effective in reducing emotional distress and had an impact on cardiac health status [3–5]. Establishing a more symptom focused therapy for depression in CHD has the potential to boost these treatment effects. Results from the multiple regression analyses underpin this assumption, because only certain depressive symptoms were associated with core cardiac symptoms and quality of life: Somatic symptoms of depression and suicidal ideations predicted NYHA class. CSS class was predicted by somatic and affective symptoms of depression. The close interplay between depressive and cardiac core symptoms could explain why studies find individual depressive symptoms to predict cardiac events and mortality [8, 53]. To understand the etiology of depression in CHD, future research should focus on the dynamic relationship between individual depressive symptoms, cardiac risk factors and a decline in cardiac health status. Results of the present study offer some of the first insights into how depressive symptoms interact with patient characteristics. To identify subgroups and tailor symptom focused interventions, it might be useful to test whether associations between individual depressive symptoms and cardiac symptoms interact with certain patient factors (e.g. age, gender, medication, atherosclerotic progression, coping style). It is now technologically possible to track individual symptoms (i.e. loss of interest), related risk behaviors (i.e. sedentary behavior), cardiac risk factors (i.e. obesity) and cardiac events over time. Such a network approach has the means to identify how maladaptive behavior interacts with depressive symptoms and increased risk of a cardiac death early on and, thus, could offer new insights for interventions [54].

### Limitations

Although the findings of the present study are based on a large, well categorized, consecutive sample of patients from three different treatment settings, factors such as disease stage, atherosclerotic progress, cardiac emergency, medication and other comorbidities may have influenced the frequency of depressive symptoms. Moreover, the fact that patients were invited to participate in a randomized-controlled trial on depression screening and patient-targeted feedback might have influenced their responses as depressed patients might have been more likely to seek help and participate. However, the average depression score in our patient population was comparable with others studies involving cardiac patients. In addition, the cross-sectional design does not allow us to determine the time course of depressive symptoms nor to draw causal interpretations based on the multiple regression analyses. In line with previous findings, results of the present study suggest that individual depressive symptoms can predict increased cardiac symptom burden and low quality of life. Still, longitudinal studies are needed to investigate the relationship between depressive symptoms, cardiac outcomes, and subjective health over time. When considering health-related quality of life, the associations between individual depressive symptoms and the EQ-5D score might have been inflated by the fact that one dimension of the EQ-5D is labeled “Anxiety/depression”. To adjust for this potential bias, future studies should incorporate questionnaires that distinguish between physical and mental quality of life. Finally, the study is based solely on self-report and no psychiatric interviews were performed. Nevertheless, the aim of this study was not to assess major depressive disorder but to investigate individual depressive symptoms. Importantly, the PHQ-9 has been shown to have reasonable psychometric properties in cardiology and in primary care.

### Conclusions

To best of our knowledge, this is the first study that reports the base rates of depressive symptoms in patients with CHD. Depressive symptoms are very common but the range of frequencies of each depressive symptom is wide. Only certain depressive symptoms are highly associated with low quality of life, impairment caused by angina pectoris and dyspnea. Taken together, these findings highlight the frequent burden of depressive symptoms in patients with CHD and their close relationship with cardiac health. Base rates of depressive symptoms offer clinicians a way to discuss individual PHQ-9 profiles with patients. This symptomatic approach to depression suggests that an efficient treatment for psychological distress in cardiac patients should not be based on a definition of a diagnosis but focus on the depressive symptoms perceived as being most burdensome by patients.

## Supporting Information

S1 Data Base rates of depressive symptoms in patients with coronary heart disease data. (CSV)

## Abbreviations

CHD: coronary heart disease
PHQ-9: Patient Health Questionnaire-9
NYHA: New York Heart Association
CCS: Canadian Cardiology Society
CI: Confidence Interval
EQ-5D: EuroQol-5D
